# The emerging role of microRNAs in bone remodeling and its therapeutic implications for osteoporosis

**DOI:** 10.1042/BSR20180453

**Published:** 2018-06-21

**Authors:** Qianyun Feng, Sheng Zheng, Jia Zheng

**Affiliations:** 1 Department of Endocrinology, Peking Unversity First Hospital, Beijing, China; 2 Graduate School, Tianjin University of Traditional Chinese Medicine, Tianjin, China; 3 Department of Spine Surgery, Tianjin Union Medical Center, Tianjin Institute of Spine, Tianjin, China

**Keywords:** bone remodeling, bone fracture, epigenetics, microRNA, osteoporosis

## Abstract

Osteoporosis, a common and multifactorial disease, is influenced by genetic factors and environments. However, the pathogenesis of osteoporosis has not been fully elucidated yet. Recently, emerging evidence suggests that epigenetic modifications may be the underlying mechanisms that link genetic and environmental factors with increased risks of osteoporosis and bone fracture. MicroRNA (miRNA), a major category of small noncoding RNA with 20–22 bases in length, is recognized as one important epigenetic modification. It can mediate post-transcriptional regulation of target genes with cell differentiation and apoptosis. In this review, we aimed to profile the role of miRNA in bone remodeling and its therapeutic implications for osteoporosis. A deeper insight into the role of miRNA in bone remodeling and osteoporosis can provide unique opportunities to develop a novel diagnostic and therapeutic approach of osteoporosis.

## Introduction

### Epidemiology of osteoporosis and bone fracture

Osteoporosis, a common and complex disease, is increasing dramatically with the aging of the population [1,2]. It is a multifactorial bone disorder with deterioration of microarchitecture and compromised bone strength, which predisposes bones to higher risks of bone fragility and bone fracture [3]. It is a chronic disease affecting both sexes and all races, exerting a strong influence on life quality, morbidity, and even mortality. It is estimated that the prevalence of osteoporosis is in more than 75 million people worldwide, and the number will increase to approximately 14 million by the year 2020 in the United States [1]. Osteoporosis-related fracture is over 1.5 million annually, and hip fracture is estimated to project to 6.3 million in 2050 [4]. Strikingly, the mortality rate is approximately 20% during the first year following a hip fracture [5]. Vertebral fractures are associated with increased risks of height loss, back pain, deformity, and mortality. It can increase the future risks of additional vertebral fractures by 5 to 10 times [6]. In the United States, direct healthcare costs of osteoporosis and its related bone fractures are estimated to be 19 billion USD per year [7]. However, as a global health concern, the condition remains severely underprevented, underdiagnosed, and undertreated.

### Bone remodeling and pathogenesis of osteoporosis

**Figure 1:**
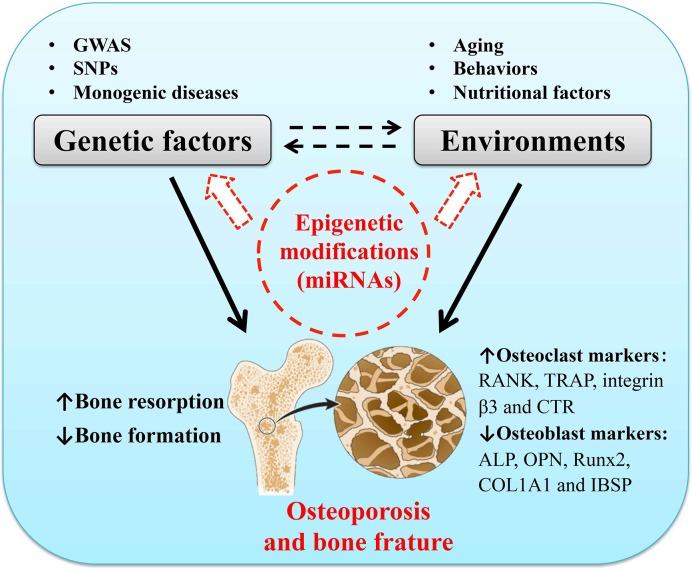
Epigenetic modifications underlying the risks of osteoporosis and bone fracture Osteoporosis is a common and complex disease with multifactorial origin that is influenced by both genes and environments. Epigenetic modifications, especially miRNAs represent a promising area to link genetics and gene expressions with the risks of osteoporosis and bone fracture; ALP, alkaline phosphatase; COL1A1, collagen, type I, α 1; CTR, calcitonin receptor; IBSP, integrin binding sialoprotein; OPN, osteopontin; RANK, the receptor activator of nuclear factor-κ B; TRAP, tartrate-resistant acid phosphatase.

The skeleton microstructure is composed of mineralized extracellular matrix and bone remodeling units, including osteocytes, osteoblasts, osteoclasts, and lining cells [1]. The function of osteoclasts and osteoblasts is critical in maintenance and remodeling of bones. Bone remodeling is a lifelong process with new bone tissues formed and mature bone tissues resorbed, also known as bone formation and bone resorption [8]. An imbalance of bone formation and bone resorption can result in metabolic bone diseases. If the process of bone resorption is faster than new bone formation, osteoporosis can finally occur [9]. Osteoporosis is a multifactorial disease that can be regulated by both genetic factors and environments. Using genome-wide association studies, numerous studies about genetic risks for osteoporosis have been performed to assess bone mineral density (BMD) as a quantitative trait [10]. It reported that more than 60 genes were related with BMD and the development of osteoporosis [11]. Several studies have identified a number of single nucleotide polymorphisms associated with a low BMD or an increased risk of fracture [12], such as vitamin D receptor gene [13], insulin-like growth factor 1 gene [14], and estrogen receptor α gene [15]. It also has demonstrated that genetic causes of monogenic bone disorders with abnormal high or low bone mass and strength can induce osteoporosis [16]. Although genetic factors are important for the development of osteoporosis and other bone diseases, it is reported that the power of genetic variables in bone remodeling is less than 3% [17]. In addition to genetic factors, behaviors (such as low level of physical activity, cigarette smoking, and caffeine intake) together with nutrients (including dietary calcium intake and vitamin D deficiency) are critical determinants of osteoporosis and bone fracture [4]. Recently, emerging evidence suggests that epigenetic modifications may be the underlying mechanisms that link genetic and environmental factors with an altered risk of osteoporosis [18,19]. A hypothetical model was tentatively proposed to illustrate the interactions among genetic factors, environments and epigenetics, and the potential mechanism underlying the role of microRNAs (miRNAs) in bone remodeling and osteoporosis (Figure 1). A deeper insight into the epigenetic mechanisms underlying bone remodeling will provide opportunities to develop a novel therapeutic approach for osteoporosis and bone fracture.

## Epigenetics and miRNAs

### What is epigenetics?

Epigenetics is the study of heritable changes in gene function—a change in phenotype without a change in genotype, which was coined by Waddington in 1942 [20]. In recent years, epigenetics has been widely accepted as an important molecular process that can regulate the activity of genome and does not involve any changes to the underlying DNA sequence [21]. It can be inherited steadily by mitosis and meiosis through cell differentiation and division between generations [22]. Epigenetics is characterized with three main features: without any alterations in the DNA sequence, heritability, and reversibility. Epigenetic regulations are involved in gene expressions that can produce permanent changes associated with cell differentiation and development [22]. There are three major categories of epigenetic modifications including DNA methylation, histone modification, and noncoding RNAs. For DNA methylation, the first genome-wide DNA methylation analysis of trabecular bone biopsies identified 241 CpG sites in 228 genes which were significantly differentially methylated between femur fracture patients and control osteoarthritis patients [24]. Zhang et al. [25] also examined histone acetylation and methylation in critical osteogenic genes during osteogenesis and found dynamic and distinct histone modifications of osteogenic genes during osteogenic differentiation. Noncoding RNAs are the latest discovered epigenetic modifications, such as miRNAs, long noncoding RNAs and circular RNA [26]. In our review, we mainly focused on the emerging role of miRNAs in bone remodeling, osteoporosis, and bone fracture.

### A glimpse at miRNAs

MiRNAs are the most studied noncoding RNAs related with bone metabolism and bone diseases. MiRNAs are a major class of small RNA molecules (approximately 20–22 nucleotides), which can decrease the expressions of target genes at post-transcriptional level [27]. It can regulate post-transcriptional gene expressions by binding to the 3′-untranslated regions (3′-UTR) of target genes, resulting in mRNA degradation and transcription inhibition [28]. Each miRNA has a number of targets, and several miRNAs can target to the same mRNA. This phenomenon indicates that the process of miRNA regulation is complex [29]. To date, over 2000 miRNAs have been identified, and up to 60% of human genome can be regulated by miRNAs [30]. MiRNAs play a critical role in the regulation of most biological processes, including cell development [31], cell differentiation, cell proliferation [32], cell cycle regulation, and metabolism [26]. Numerous evidence demonstrated that miRNAs can regulate bone remodeling and the development of osteoporosis and bone fracture [33].

## miRNA and its role in the process of bone remodeling

### miRNAs and osteoblast differentiation

**Figure 2:**
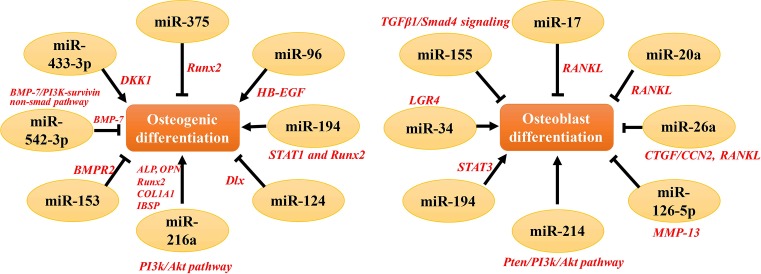
MiRNAs and their roles in osteoblast and osteoclast differentiation A number of miRNAs have been clearly found and deeply involved in the regulation of osteoblast and osteoclast differentiation, by targeting to bone-related genes and different signaling pathways; ALP, alkaline phosphatase; BMP-7, bone morphogenetic protein-7; BMPR2, bone morphogenetic protein receptor type II; COL1A1, collagen, type I, α 1; CTGF/CCN2, connective tissue growth factor/CCN family 2; DKK1, Dickkopf-1; IBSP, integrin binding sialoprotein; LGR4, leucine-rich repeat-containing G-protein-coupled receptor 4; MMP-13, matrix metalloproteinase-13; OPN, osteopontin; PI3K, phosphatidylinositol 3 kinase; RANKL, the receptor activator of nuclear factor-Κ B ligand; Runx2, runt-related transcription factor 2; STAT3, signal transducer and activator of transcription 3; TGFβ1, transforming growth factor β1.

In the last decade, a large number of miRNAs have been clearly found and deeply involved in the regulation of bone remodeling, including bone resorption and formation [34–36]. The role of miRNAs in both osteoclasts and osteoblasts growth and differentiation has been largely investigated [37]. Osteoblast differentiation is an important process of bone homeostasis. Increasing studies indicated that miRNAs can regulate the biological process of osteoblast differentiation [33]. Several miRNAs can target the 3′-UTR of Runt-related transcription factor 2 (Runx2), the one important molecule that can regulate bone-related gene expressions [38]. Overexpression of miR-375 inhibited osteogenic differentiation by targeting Runx2, with decreased activity of several critical osteoblast markers, such as alkaline phosphatase (ALP), osteocalcin, and IBSP [39]. It also showed that miR-96 promoted osteogenic differentiation by suppressing heparin-binding epidermal growth factor-like growth factor (HB-EGF)–EGF receptor signaling in osteoblastic cells [40]. Li et al. [41] found that miR-194 regulated osteoblast differentiation through modulating signal transducer and activator of transcription 1 (STAT1)-mediated Runx2 nuclear translocation. Some miRNAs have been profiled to be able to promote and suppress distinct signaling pathway related with osteogenic differentiation. In mesenchymal stem cells (MSC), miR-124 could inhibit osteogenic differentiation and bone formation by targeting Dlx transcription factors, including Dlx5, Dlx3, and Dlx2 genes [42]. MiR-216a rescued osteogenesis, enhanced osteoblast differentiation and bone formation, by regulating c-Cbl-mediated phosphatidylinositol 3 kinase (PI3K)/protein kinase B/serine-threonine protein kinase (AKT) pathway [43]. In a human MSC, miRNA-153 could suppress osteogenic differentiation, with targeting bone morphogenetic protein receptor type II (BMPR2) [44]. MiR-542-3p suppressed osteoblast cell differentiation and proliferation, targeted bone morphogenetic protein-7 (BMP-7) signaling and then inhibited bone formation [45]. Dickkopf-1 (DKK1), as an important biomarker for osteoporosis, is an antagonist of WNT signaling pathway. One recent study showed that miRNA-433-3p promoted osteoblast differentiation with DKK1 as the target gene [46]. These data suggest that miRNAs play a significant role in the process of osteoblast differentiation targeting major genes and signaling pathways related with osteogenic differentiation. The relevant studies showing the role of miRNAs in osteoblast differentiation were summarized in Table 1 and Figure 2.

**Table 1:** Summary of the relevant studies showing the role of miRNAs in osteoblast differentiation

MiRNA ID	Cell types	Target genes and pathways	Effects on bone remodeling	Year	References
miR-375	C2C12 cell	Runx2	Inhibited osteogenic differentiation	2015	Du et al. [39]
miR-96	MC3T3-E1 cells and mouse MSCs	Heparin-binding EGF-like growth factor (HB-EGF)	Promoted osteogenic differentiation	2014	Yang et al. [40]
miR-194	Bone mesenchymal stem cells (BMSCs)	STAT1 and Runx2	Promoted osteoblast differentiation	2015	Li et al. [41]
miR-124	Human and mouse MSCs, MC3T3-E1 cells, and C2C12 cells	Dlx transcription factors: Dlx5, Dlx3, and Dlx2	Inhibited osteogenic differentiation	2015	Qadir et al [42].
miR-216a	Human adipose-derived MSCs (hAMSCs)	Osteoblast marker genes (ALP, OPN, Runx2, COL1A1 and IBSP), and c-Cbl-mediated PI3K/AKT pathway	Promoted osteoblast differentiation and enhanced bone formation	2015	Li et al. [43]
miR-153	Human mesenchymal stem cells (hMSCs)	BMPR2	Suppressed osteogenic differentiation	2015	Cao et al. [44]
miR-542-3p	Human osteoblast cells	BMP-7 and BMP-7/PI3K-survivin non-Smad pathway	Suppressed osteoblast cell proliferation and differentiation	2014	Kureel et al. [45]
MiR-433-3p	Rat bone marrow derived osteoblasts	DKK1	Promoted osteoblast differentiation	2017	Tang et al. [46]

Abbreviations: ALP, alkaline phosphatase; BMP-7, bone morphogenetic protein-7; BMPR2, bone morphogenetic protein receptor type II; COL1A1, collagen, type I, α 1; DKK1, Dickkopf-1; IBSP, integrin binding sialoprotein; MSC, mesenchymal stem cell; OPN, osteopontin; PI3K, phosphatidylinositol 3 kinase; Runx2, runt-related transcription factor 2; STAT1, signal transducer and activator of transcription 1.

### MiRNAs and osteoclast differentiation

Apart from an osteoblast differentiation, the expression pattern of miRNAs related with the osteoclast differentiation has also been explored. The ligand–receptor system of receptor activator of nuclear factor-kappa B (RANK)/RANK ligand (RANKL)/osteoprotegerin (OPG) is a key component in bone tissue metabolism, which can regulate osteoclasts differentiation and osteolysis. MiRNA-17/20a could target RANKL and inhibit osteoclast differentiation induced by glucocorticoid in osteoblast cells [47]. MiRNA-26a mimic ectopic expression could attenuate osteoclast and actin-ring formation in osteoclast precursor cells, with decreased expression of connective tissue growth factor/CCN family 2 (CTGF/CCN2). Overexpression of miRNA-26a inhibitor promoted RANKL-induced osteoclast formation and function [48]. In addition to RANK/RANKL/OPG system, miRNAs also can regulate other signaling pathways related with osteoclast differentiation. Wu et al. [49] found that miR-126-5p overexpression could inhibit osteoclast differentiation and decrease osteolysis formation in the stromal cells of a giant cell tumor. In bone marrow monocytes (BMMs), miRNA-214 overexpression promoted osteoclastogenesis by PI3K/Akt pathway, with phosphatase and tensin homolog (Pten) as the potential target [50]. Liu et al. [51] identified a novel miRNA (miR-9718) in primary mouse osteoclasts that could enhance osteoclast differentiation, with post-transcriptional repression of protein inhibitor of activated STAT3 (PIAS3). Recent studies found that miR-34c significantly promoted osteoclast differentiation targeting 3′-untranslated region of leucine-rich repeat-containing G-protein-coupled receptor 4 (LGR4) [52], while miR-155 mediated its suppressive effect on osteoclast differentiation by targeting transforming growth factor β1 (TGFβ1)/Smad4 signaling pathway [53]. Taken together, the aforementioned studies demonstrated that miRNAs can play a critical role in osteoclast differentiation. The relevant evidence was summarized in Table 2 and Figure 2.

**Table 2:** Summary of the relevant studies showing the role of miRNAs in osteoclast differentiation

MiRNA ID	Cell types	Target genes and pathways	Effects on bone remodeling	Year	References
miR- 17/20a	Osteoblast cells	RANKL	Inhibited glucocorticoid-induced osteoclast differentiation and function	2014	Shi et al. [47]
miR-26a	Osteoclast precursor cells	CTGF/CCN2, RANKL	Attenuated osteoclast formation, actin-ring formation, and bone resorption	2015	Kim et al. [48]
miR- 126-5p	The stromal cells of giant cell tumor	MMP-13	Inhibited osteoclast differentiation	2014	Wu et al. [49]
miR-214	BMMs	Pten/PI3k/Akt pathway	Promoted osteoclastogenesis	2015	Zhao et al. [50]
miR-194	Primary mouse osteoclasts	STAT3	Promoted osteoclast differentiation	2014	Liu et al. [51]
miR-34	Osteoclast precursors	LGR4	Promoted osteoclast differentiation	2017	Cong et al. [52]
miR-155	Bone marrow-derived macrophages	TGFβ1/Smad4 signaling	Inhibited osteoclast differentiation	2017	Zhao et al. [53]

Abbreviations: CTGF/CCN2, connective tissue growth factor/CCN family 2; LGR4, leucine-rich repeat-containing G-protein-coupled receptor 4; MMP-13, matrix metalloproteinase-13; PI3K, phosphatidylinositol 3 kinase; RANKL, the receptor activator of nuclear factor-kappa B ligand; STAT3, signal transducer and activator of transcription 3; TGFβ1, transforming growth factor β1.

## Circulating miRNAs in osteoporosis and bone fracture

It reported that miRNAs can be secreted by different cells and have been discovered in the bloodstream and bodily fluids [54–58]. Circulating miRNAs have the potential to be utilized as novel biomarkers for the early diagnosis, treatment and prognosis of several diseases, such as cancer [59], cardiovascular diseases [60,61], obesity [62], and diabetes mellitus [63]. Recent studies demonstrated that serum miRNA levels were markedly up-regulated in patients with osteoporotic fractures and could impact osteogenic differentiation [56]. Nine miRNAs, including miR-93, miR-24, miR-23a, miR-124a, miR-122a, miR-21, miR-125b, miR-100, and miR-148a were significantly increased in the serum of 30 patients with osteoporosis, compared with 30 nonosteoporotic controls [64]. Li et al. [65] detected three miRNAs (miR-133a, miR-21, and miR-146) levels in the blood of 120 postmenopausal women and found that plasma miR-21 was decreased and miR-133a was increased in patients with osteoporosis and osteopenia, compared with the normal group according to T-scores of BMD. Weilner et al. [66] aimed to profile whether the expression of circulating miRNAs were variable in patients with newly osteoporotic fracture. Of 175 miRNAs in serum samples, the expression levels of six miRNAs (miR-133b, miR-22-3p, miR-10a-5p, miR-10b-5p, let-7g-5p, and miR-328-3p) were significantly related with bone fracture. These miRNAs were subsequently analyzed and further validated in a cohort with a larger sample size. Panach et al. [78] measured the expression levels of 179 serum miRNAs in osteoporotic women with fractures, and three miRNAs (miR-122-5p, miR-21-5p, and miR-125b-5p) were significantly up-regulated and indicated as valuable biomarkers in bone fracture. MiR-148a was reported to be increased in plasma in postmenopausal women with osteoporosis, and serum miR-148a level was correlated with clinical parameters of bone quality and quantity [67]. In a subsequent analysis, Kocijan et al. [68] assessed circulating miRNA signatures in male and female subjects with idiopathic or postmenopausal osteoporotic fractures and found that eight miRNAs were confirmed to be excellent discriminators of fractures regardless of age and gender. The listed studies reveal an important role for circulating miRNAs in osteoporotic patients. As the most abundant RNA species, miRNAs can be easily detected in circulation. It suggested that miRNAs can be utilized as novel biomarkers for early diagnosis and treatment, and it may be a potential target for medicine development. However, more comprehensive studies with larger samples and longer follow-up are warranted to investigate the significance of circulating miRNAs in osteoporosis and bone frature.

## MiRNAs and its therapeutic implications for osteoporosis

Generally, it was deemed that the process of epigenetic regulation was static. However, this viewpoint has been altered and epigenetic modifications, including miRNAs, were perceived as dynamic and even reversible. It has been demonstrated that miRNAs are extremely attractive targets for therapeutic regulation in several diseases, such as brain tumors [69], gastrointestinal cancers [70], and cardiovascular diseases [71]. Compounds targeting specific miRNAs are currently utilized for the treatment of lung cancer. More specifically, chemically synthesized miR-34a mimic was administered intratumorally or intravenously by tail vein injections in mice (100 μg), which could restore a loss of function in cancer that drives a therapeutic response of lung cancer [72]. Silencing of miR-103/107 targeting caveolin-1 could lead to improved glucose homeostasis and insulin sensitivity and type II diabetes [73]. Some preliminary studies have showed that miRNAs play a critical role in the treatment of osteoporosis and bone fracture. Resveratrol, as a polyphenolic phytoestrogen with osteoinductive and osteogenic properties, could prevent osteoporosis in ovariectomized rats by suppressing the expression of miR-338-3p and increasing the expression of Runx2 [74]. Recently, it indicated that miR-365 could ameliorate osteogenesis suppression in MC3T3-E1 cells by targeting histone deacetylase 4 (HDAC4) [75]. In a glucocorticoid-induced osteoporosis C57BL/6J mice model, Li et al. [76] showed that curcumin improved bone microarchitecture by activating miRNA-365 targeting matrix metalloproteinase-9 (MMP-9). These two studies both suggest that miR-365 may be an important molecular regulating glucocorticoid-induced osteoporosis. Zhang et al. [77] demonstrated that RANKL was directly regulated by miR-338-3p and reintroduction of RANKL could reverse the inhibitory effects of miR-338-3p on osteoclast formation and bone resorption. However, the studies about therapeutic effects of miRNAs were limited, the specific doses and time point for miRNAs treatment are uncertain, and more preclinical studies are warranted. Since miRNAs are the most abundant RNA species to be found in circulation, quantification of their expression may be used as biomarker for early therapeutic effects and prognostic purposes of miRNAs-based treatment. It is speculated that the uptake of miRNA mimics is safe, which has no effect on normal cells because pathways regulated by the miRNA mimics are already activated by the endogenous miRNA in the cells [72]. However, the promiscuity of miRNAs should be addressed. One certain miRNA may have hundreds of transcription targets, thus the inhibition could lead to unwanted collateral effects. The current predictions by TargetScan, PicTar, EMBL, and ElMMo have a high degree of overlap because they all require stringent seed pairing. However, they are not 100% identical, increasing the difficulties of target predictions. Thus, this is maybe a major limitation to the application and development of therapies of miRNAs.

## Conclusions

In summary, miRNA plays a critical role in the process of bone remodeling, including both osteoblast differentiation and osteoblast differentiation. Serum circulating miRNAs were detected and profiled in patients with osteoporosis and bone fracture. Furthermore, increasing evidences show that epigenetic modifications are not static, and are dynamic and even reversible. Thus, a deeper understanding of the role of miRNAs in osteoporosis and bone fracture can inspire critical implications for the early diagnosis and prevention of osteoporosis. It also can provide unique opportunities to develop novel therapeutic approaches of osteoporosis and its related bone fracture.

## Abbreviations

ALP: alkaline phosphatase
BMD: bone mineral density
BMM: bone marrow monocyte
BMP-7: bone morphogenetic protein-7
BMPR2: bone morphogenetic protein receptor type II
CCN2: CCN family 2
CpG: cytosines following by guanine
CTGF: connective tissue growth factor
DKK1: Dickkopf-1
HDAC4: histone deacetylase 4
LGR4: leucine-rich repeat-containing G-protein-coupled receptor 4
miRNA: microRNA
MMP-9: matrix metalloproteinase-9
MSC: mesenchymal stem cells
OPG: osteoprotegerin
Pten: phosphatase and tensin homolog
RANK: receptor activator of nuclear factor-kappa B
RANKL: RANK ligand
Runx2: Runt-related transcription factor 2
STAT1: signal transducer and activator of transcription 1
TGFβ1: transforming growth factor β1

## References

[1] Schuiling K.D., Robinia K. and Nye R. (2011) Osteoporosis update. J. Midwifery Womens Health 56, 615–627 10.1111/j.1542-2011.2011.00135.x 22060222

[2] Tucker K.L. (2003) Dietary intake and bone status with aging. Curr. Pharm. Des. 9, 2687–2704 10.2174/1381612033453613 14529541

[3] (1993) Consensus development conference: diagnosis, prophylaxis, and treatment of osteoporosis. Am. J. Med. 94, 646–650 10.1016/0002-9343(93)90218-E 8506892

[4] Lane N.E. (2006) Epidemiology, etiology, and diagnosis of osteoporosis. Am. J. Obstet. Gynecol. 194, S3–S11 10.1016/j.ajog.2005.08.047 16448873

[5] Cooper C., Atkinson E.J., Jacobsen S.J., O’Fallon W.M. and Melton L.J. III (1993) Population-based study of survival after osteoporotic fractures. Am. J. Epidemiol. 137, 1001–1005 10.1093/oxfordjournals.aje.a116756 8317445

[6] Wustrack R., Seeman E., Bucci-Rechtweg C., Burch S., Palermo L. and Black D.M. (2012) Predictors of new and severe vertebral fractures: results from the HORIZON Pivotal Fracture Trial. Osteoporosis Int. 23, 53–58 10.1007/s00198-011-1664-4 21691843

[7] The Global Burden of Osteoporosis. International Osteoporosis Foundation, www.iofbonehealth.org (accessed 2016–02–09)

[8] Sobacchi C., Schulz A., Coxon F.P., Villa A. and Helfrich M.H. (2013) Osteopetrosis: genetics, treatment and new insights into osteoclast function. Nat. Rev. Endocrinol. 9, 522–536 10.1038/nrendo.2013.137 23877423

[9] Wu S., Liu Y., Zhang L., Han Y., Lin Y. and Deng H.W. (2013) Genome-wide approaches for identifying genetic risk factors for osteoporosis. Genome Med. 5, 44 10.1186/gm448 23731620 PMC3706967

[10] Mori S. (2016) Genome-wide association study for Osteoporosis. Clin. Calcium 26, 537–543 27013623

[11] Kung A.W. and Huang Q.Y. (2007) Genetic and environmental determinants of osteoporosis. J. Musculoskeletal Neuronal Interact. 7, 26–32 17396003

[12] Ralston S.H. (2005) Genetic determinants of osteoporosis. Curr. Opin. Rheumatol. 17, 475–479 10.1097/01.bor.0000166385.62851.92 15956846

[13] Thakkinstian A., D’Este C., Eisman J., Nguyen T. and Attia J. (2004) Meta-analysis of molecular association studies: vitamin D receptor gene polymorphisms and BMD as a case study. J. Bone Miner. Res. 19, 419–428 10.1359/JBMR.0301265 15040830

[14] Rivadeneira F., Houwing-Duistermaat J.J., Vaessen N., Vergeer-Drop J.M., Hofman A., Pols H.A. (2003) Association between an insulin-like growth factor I gene promoter polymorphism and bone mineral density in the elderly: the Rotterdam Study. J. Clin. Endocrinol. Metab. 88, 3878–3884 10.1210/jc.2002-021813 12915683

[15] Ioannidis J.P., Stavrou I., Trikalinos T.A., Zois C., Brandi M.L., Gennari L. (2002) Association of polymorphisms of the estrogen receptor alpha gene with bone mineral density and fracture risk in women: a meta-analysis. J. Bone Miner. Res. 17, 2048–2060 10.1359/jbmr.2002.17.11.2048 12412813

[16] Boudin E. and Van Hul W. (2017) Mechanisms in endocrinology: genetics of human bone formation. Eur. J. Endocrinol. 177, R69–R83 10.1530/EJE-16-0990 28381451

[17] Ralston S.H. (1998) Do genetic markers aid in risk assessment? Osteoporosis Int. 8, S37–S42 9682796

[18] Yasui T., Hirose J., Aburatani H. and Tanaka S. (2011) Epigenetic regulation of osteoclast differentiation. Ann. N. Y. Acad. Sci. 1240, 7–13 10.1111/j.1749-6632.2011.06245.x 22172033

[19] Ghayor C. and Weber F.E. (2016) Epigenetic regulation of bone remodeling and its impacts in osteoporosis. Int. J. Mol. Sci. 17, 1–14, 10.3390/ijms17091446 27598138 PMC5037725

[20] Waddington C.H. (2012) The epigenotype. 1942. Int. J. Epidemiol. 41, 10–13 10.1093/ije/dyr184 22186258

[21] Skinner M.K., Manikkam M. and Guerrero-Bosagna C. (2010) Epigenetic transgenerational actions of environmental factors in disease etiology. Trends Endocrinol. Metab. 21, 214–222 10.1016/j.tem.2009.12.007 20074974 PMC2848884

[22] Holliday R. (2006) Epigenetics a historical overview. Epigenetics 1, 76–80 10.4161/epi.1.2.2762 17998809

[23] Reference deleted

[24] Delgado-Calle J., Fernandez A.F., Sainz J., Zarrabeitia M.T., Sanudo C., Garcia-Renedo R. (2013) Genome-wide profiling of bone reveals differentially methylated regions in osteoporosis and osteoarthritis. Arthritis Rheum. 65, 197–205 10.1002/art.37753 23124911

[25] Zhang Y.X., Sun H.L., Liang H., Li K., Fan Q.M. and Zhao Q.H. (2015) Dynamic and distinct histone modifications of osteogenic genes during osteogenic differentiation. J. Biochem. 158, 445–457 26078467 10.1093/jb/mvv059

[26] Aguilera O., Fernandez A.F., Munoz A. and Fraga M.F. (2010) Epigenetics and environment: a complex relationship. J. Appl. Physiol. 109, 243–251 10.1152/japplphysiol.00068.2010 20378707

[27] Pepin G. and Gantier M.P. (2016) microRNA decay: refining microRNA regulatory activity. MicroRNA 5, 167–174 27804865 10.2174/2211536605666161027165915

[28] Reddy M.A. and Natarajan R. (2011) Epigenetic mechanisms in diabetic vascular complications. Cardiovasc. Res. 90, 421–429 10.1093/cvr/cvr024 21266525 PMC3096305

[29] Stefani G. and Slack F. (2006) MicroRNAs in search of a target. Cold Spring Harb. Symp. Quant. Biol. 71, 129–134 10.1101/sqb.2006.71.032 17381288

[30] Sayed D. and Abdellatif M. (2011) MicroRNAs in development and disease. Physiol. Rev. 91, 827–887 10.1152/physrev.00006.2010 21742789

[31] Wienholds E. and Plasterk R.H. (2005) MicroRNA function in animal development. FEBS Lett. 579, 5911–5922 10.1016/j.febslet.2005.07.070 16111679

[32] Oakley E.J. and Van Zant G. (2007) Unraveling the complex regulation of stem cells: implications for aging and cancer. Leukemia 21, 612–621 10.1038/sj.leu.2404530 17252019

[33] Husain A. and Jeffries M.A. (2017) Epigenetics and bone remodeling. Curr. Osteoporos. Rep., 15, 450–458 10.1007/s11914-017-0391-y 28808893 PMC5710824

[34] Nugent M. (2017) MicroRNAs and fracture healing. Calcif. Tissue Int. 101, 355–361 10.1007/s00223-017-0296-x 28589206

[35] Taipaleenmaki H. (2018) Regulation of bone metabolism by microRNAs. Curr. Osteoporos. Rep. 16, 1–12 10.1007/s11914-018-0417-0 29335833

[36] Valenti M.T., Dalle Carbonare L. and Mottes M. (2018) Role of microRNAs in progenitor cell commitment and osteogenic differentiation in health and disease (Review). Int. J. Mol. Med. 41, 2441–2449 29393379 10.3892/ijmm.2018.3452

[37] Ge D.W., Wang W.W., Chen H.T., Yang L. and Cao X.J. (2017) Functions of microRNAs in osteoporosis. Eur. Rev. Med. Pharmacol. Sci. 21, 4784–4789 29164586

[38] Zhang Y., Xie R.L., Croce C.M., Stein J.L., Lian J.B., van Wijnen A.J. (2011) A program of microRNAs controls osteogenic lineage progression by targeting transcription factor Runx2. Proc. Natl. Acad. Sci. U.S.A. 108, 9863–9868 10.1073/pnas.1018493108 21628588 PMC3116419

[39] Du F., Wu H., Zhou Z. and Liu Y.U. (2015) microRNA-375 inhibits osteogenic differentiation by targeting runt-related transcription factor 2. Exp. Therapeutic Med. 10, 207–212 10.3892/etm.2015.2477 PMC4487083 26170936

[40] Yang M., Pan Y. and Zhou Y. (2014) miR-96 promotes osteogenic differentiation by suppressing HBEGF-EGFR signaling in osteoblastic cells. FEBS Lett. 588, 4761–4768 10.1016/j.febslet.2014.11.008 25451232

[41] Li J., He X., Wei W. and Zhou X. (2015) MicroRNA-194 promotes osteoblast differentiation via downregulating STAT1. Biochem. Biophys. Res. Commun. 460, 482–488 10.1016/j.bbrc.2015.03.059 25797619

[42] Qadir A.S., Um S., Lee H., Baek K., Seo B.M., Lee G. (2015) miR-124 negatively regulates osteogenic differentiation and in vivo bone formation of mesenchymal stem cells. J. Cell. Biochem. 116, 730–742 10.1002/jcb.25026 25424317

[43] Li H., Li T., Fan J., Li T., Fan L., Wang S. (2015) miR-216a rescues dexamethasone suppression of osteogenesis, promotes osteoblast differentiation and enhances bone formation, by regulating c-Cbl-mediated PI3K/AKT pathway. Cell Death Differ. 22, 1935–1945 10.1038/cdd.2015.99 26206089 PMC4816120

[44] Cao Y., Lv Q. and Lv C. (2015) MicroRNA-153 suppresses the osteogenic differentiation of human mesenchymal stem cells by targeting bone morphogenetic protein receptor type II. Int. J. Mol. Med. 36, 760–766 10.3892/ijmm.2015.2275 26151470

[45] Kureel J., Dixit M., Tyagi A.M., Mansoori M.N., Srivastava K., Raghuvanshi A. (2014) miR-542-3p suppresses osteoblast cell proliferation and differentiation, targets BMP-7 signaling and inhibits bone formation. Cell Death Dis. 5, e1050 10.1038/cddis.2014.4 24503542 PMC3944264

[46] Tang X., Lin J., Wang G. and Lu J. (2017) MicroRNA-433-3p promotes osteoblast differentiation through targeting DKK1 expression. PLoS ONE 12, e0179860 10.1371/journal.pone.0179860 28628652 PMC5476290

[47] Shi C., Qi J., Huang P., Jiang M., Zhou Q., Zhou H. (2014) MicroRNA-17/20a inhibits glucocorticoid-induced osteoclast differentiation and function through targeting RANKL expression in osteoblast cells. Bone 68, 67–75 10.1016/j.bone.2014.08.004 25138550

[48] Kim K., Kim J.H., Kim I., Lee J., Seong S., Park Y.W. (2015) MicroRNA-26a regulates RANKL-induced osteoclast formation. Mol. Cells 38, 75–80 25518928 10.14348/molcells.2015.2241 PMC4314121

[49] Wu Z., Yin H., Liu T., Yan W., Li Z., Chen J. (2014) MiR-126-5p regulates osteoclast differentiation and bone resorption in giant cell tumor through inhibition of MMP-13. Biochem. Biophys. Res. Commun. 443, 944–949 10.1016/j.bbrc.2013.12.075 24360951

[50] Zhao C., Sun W., Zhang P., Ling S., Li Y., Zhao D. (2015) miR-214 promotes osteoclastogenesis by targeting Pten/PI3k/Akt pathway. RNA Biol. 12, 343–353 10.1080/15476286.2015.1017205 25826666 PMC4615895

[51] Liu T., Qin A.P., Liao B., Shao H.G., Guo L.J., Xie G.Q. (2014) A novel microRNA regulates osteoclast differentiation via targeting protein inhibitor of activated STAT3 (PIAS3). Bone 67, 156–165 10.1016/j.bone.2014.07.004 25019593

[52] Cong F., Wu N., Tian X., Fan J., Liu J., Song T. (2017) MicroRNA-34c promotes osteoclast differentiation through targeting LGR4. Gene 610, 1–8 10.1016/j.gene.2017.01.028 28130056

[53] Zhao H., Zhang J., Shao H., Liu J., Jin M., Chen J. (2017) Transforming growth factor beta1/Smad4 signaling affects osteoclast differentiation via regulation of miR-155 expression. Mol. Cells 40, 211–221 28359146 10.14348/molcells.2017.2303 PMC5386959

[54] Jimenez-Ortega R.F., Ramirez-Salazar E.G., Parra-Torres A.Y., Munoz-Montero S.A., Rangel-Escareno C., Salido-Guadarrama I. (2017) Identification of microRNAs in human circulating monocytes of postmenopausal osteoporotic Mexican-Mestizo women: a pilot study. Exp. Ther. Med. 14, 5464–5472 29285077 10.3892/etm.2017.5260 PMC5740757

[55] Jung H.J. and Suh Y. (2014) Circulating miRNAs in ageing and ageing-related diseases. J. Genetics Genom. = Yi chuan xue bao 41, 465–472 10.1016/j.jgg.2014.07.003 PMC4354804 25269672

[56] Hackl M., Heilmeier U., Weilner S. and Grillari J. (2016) Circulating microRNAs as novel biomarkers for bone diseases - complex signatures for multifactorial diseases? Mol. Cell. Endocrinol. 432, 83–95 10.1016/j.mce.2015.10.015 26525415

[57] Qi Z., Liu W. and Lu J. (2016) The mechanisms underlying the beneficial effects of exercise on bone remodeling: roles of bone-derived cytokines and microRNAs. Prog. Biophys. Mol. Biol. 122, 131–139 10.1016/j.pbiomolbio.2016.05.010 27179638

[58] Li Z., Jiang C., Li X., Wu W.K.K., Chen X., Zhu S. (2018) Circulating microRNA signature of steroid-induced osteonecrosis of the femoral head. Cell Prolif. 51, 1–13, 10.1111/cpr.12418 PMC6528947 29205600

[59] Schou J.V., Johansen J.S., Nielsen D. and Rossi S. (2016) Circulating microRNAs as prognostic and predictive biomarkers in patients with colorectal cancer. Non-coding RNA 2, 10.3390/ncrna2020005 29657263 PMC5831904

[60] Bayoumi A.S., Aonuma T., Teoh J.P., Tang Y.L. and Kim I.M. (2018) Circular noncoding RNAs as potential therapies and circulating biomarkers for cardiovascular diseases. Acta Pharmacol. Sin. 10.1038/aps.2017.196 29565037 PMC6289320

[61] Pereira-da-Silva T., Coutinho Cruz M., Carrusca C., Cruz Ferreira R., Napoleao P. and Mota Carmo M. (2018) Circulating microRNA profiles in different arterial territories of stable atherosclerotic disease: a systematic review. Am. J. Cardiovasc. Dis. 8, 1–13 29531852 PMC5840275

[62] Zaiou M., El Amri H. and Bakillah A. (2018) The clinical potential of adipogenesis and obesity-related microRNAs. Nutrition, Metab. Cardiovasc. Dis. 28, 91–111 10.1016/j.numecd.2017.10.015 29170059

[63] Zhang Y., Sun X., Icli B. and Feinberg M.W. (2017) Emerging roles for microRNAs in diabetic microvascular disease: novel targets for therapy. Endocr. Rev. 2017, 1–22 10.1210/er.2016-1122.2017.1.test PMC5460677 28323921

[64] Seeliger C., Karpinski K., Haug A.T., Vester H., Schmitt A., Bauer J.S. (2014) Five freely circulating miRNAs and bone tissue miRNAs are associated with osteoporotic fractures. J. Bone Miner. Res. 1728 10.1002/jbmr.2175 24431276

[65] Li H., Wang Z., Fu Q. and Zhang J. (2014) Plasma miRNA levels correlate with sensitivity to bone mineral density in postmenopausal osteoporosis patients. Biomarkers 19, 553–556 10.3109/1354750X.2014.935957 25231354

[66] Weilner S., Skalicky S., Salzer B., Keider V., Wagner M., Hildner F. (2015) Differentially circulating miRNAs after recent osteoporotic fractures can influence osteogenic differentiation. Bone 79, 43–51 10.1016/j.bone.2015.05.027 26026730

[67] Bedene A., Mencej Bedrac S., Jese L., Marc J., Vrtacnik P., Prezelj J. (2016) MiR-148a the epigenetic regulator of bone homeostasis is increased in plasma of osteoporotic postmenopausal women. Wien. Klin. Wochenschr. 128, 519–526 10.1007/s00508-016-1141-3 27900532

[68] Kocijan R., Muschitz C., Geiger E., Skalicky S., Baierl A., Dormann R. (2016) Circulating microRNA signatures in patients with idiopathic and postmenopausal osteoporosis and fragility fractures. J. Clin. Endocrinol. Metab. 101, 4125–4134 10.1210/jc.2016-2365 27552543

[69] Anthiya S., Griveau A., Loussouarn C., Baril P., Garnett M., Issartel J.P. (2018) MicroRNA-based drugs for brain tumors. Trends Cancer 4, 222–238 10.1016/j.trecan.2017.12.008 29506672

[70] Merhautova J., Demlova R. and Slaby O. (2016) MicroRNA-based therapy in animal models of selected gastrointestinal cancers. Front. Pharmacol. 7, 329 10.3389/fphar.2016.00329 27729862 PMC5037200

[71] Mellis D. and Caporali A. (2018) MicroRNA-based therapeutics in cardiovascular disease: screening and delivery to the target. Biochem. Soc. Trans. 46, 11–21 10.1042/BST20170037 29196609

[72] Wiggins J.F., Ruffino L., Kelnar K., Omotola M., Patrawala L., Brown D. (2010) Development of a lung cancer therapeutic based on the tumor suppressor microRNA-34. Cancer Res. 70, 5923–5930 10.1158/0008-5472.CAN-10-0655 20570894 PMC2913706

[73] Trajkovski M., Hausser J., Soutschek J., Bhat B., Akin A., Zavolan M. (2011) MicroRNAs 103 and 107 regulate insulin sensitivity. Nature 474, 649–653 10.1038/nature10112 21654750

[74] Guo D.W., Han Y.X., Cong L., Liang D. and Tu G.J. (2015) Resveratrol prevents osteoporosis in ovariectomized rats by regulating microRNA-338-3p. Mol. Med. Rep. 12, 2098–2106 10.3892/mmr.2015.3581 25845653

[75] Xu D., Gao Y., Hu N., Wu L. and Chen Q. (2017) miR-365 ameliorates dexamethasone-induced suppression of osteogenesis in MC3T3-E1 cells by targeting HDAC4. Int. J. Mol. Sci. 18, 10.3390/ijms18050977 PMC5454890 28471397

[76] Li G., Bu J., Zhu Y., Xiao X., Liang Z. and Zhang R. (2015) Curcumin improves bone microarchitecture in glucocorticoid-induced secondary osteoporosis mice through the activation of microRNA-365 via regulating MMP-9. Int. J. Clin. Exp. Pathol. 8, 15684–15695 26884838 PMC4730051

[77] Zhang X.H., Geng G.L., Su B., Liang C.P., Wang F. and Bao J.C. (2016) MicroRNA-338-3p inhibits glucocorticoid-induced osteoclast formation through RANKL targeting. Genet. Mol. Res. 15, 10.4238/gmr.15037674 27706599

[78] Panach L., Mifsut D., Tarín JJ., Cano A. and García-Pérez MÁ. (2015) Serum Circulating MicroRNAs as Biomarkers of Osteoporotic Fracture. Calcif. Tissue Int. 97, 495–505 10.1007/s00223-015-0036-z 26163235

